# Regulation of Trabecular Meshwork Cell Contraction and Intraocular Pressure by miR-200c

**DOI:** 10.1371/journal.pone.0051688

**Published:** 2012-12-14

**Authors:** Coralia Luna, Guorong Li, Jianyong Huang, Jianming Qiu, Jing Wu, Fan Yuan, David L. Epstein, Pedro Gonzalez

**Affiliations:** 1 Department of Ophthalmology, Duke University, Durham, North Carolina, United States of America; 2 Department of Biomedical Engineering, Duke University, Durham, North Carolina, United States of America; Hanson Institute, Australia

## Abstract

Lowering intraocular pressure (IOP) delays or prevents the loss of vision in primary open-angle glaucoma (POAG) patients with high IOP and in those with normal tension glaucoma showing progression. Abundant evidence demonstrates that inhibition of contractile machinery of the trabecular meshwork cells is an effective method to lower IOP. However, the mechanisms involved in the regulation of trabecular contraction are not well understood. Although microRNAs have been shown to play important roles in the regulation of multiple cellular functions, little is known about their potential involvement in the regulation of IOP. Here, we showed that miR-200c is a direct postranscriptional inhibitor of genes relevant to the physiologic regulation of TM cell contraction including the validated targets Zinc finger E-box binding homeobox 1 and 2 (ZEB1 and ZEB2), and formin homology 2 domain containing 1 (FHOD1), as well as three novel targets: lysophosphatidic acid receptor 1 (LPAR1/EDG2), endothelin A receptor (ETAR), and RhoA kinase (RHOA). Consistently, transfection of TM cells with miR-200c resulted in strong inhibition of contraction in collagen populated gels as well as decreased cell traction forces exerted by individual TM cells. Finally, delivery of miR-200c to the anterior chamber of living rat eyes resulted in a significant decrease in IOP, while inhibition of miR-200c using an adenoviral vector expressing a molecular sponge led to a significant increase in IOP. These results demonstrate for the first time the ability of a miRNA to regulate trabecular contraction and modulate IOP *in vivo*, making miR-200c a worthy candidate for exploring ways to alter trabecular contractility with therapeutic purposes in glaucoma.

## Introduction

The trabecular meshwork (TM) and Schlemm’s Canal (SC) constitute the major route of aqueous outflow from the eye, and is the locus of increased resistance responsible for the abnormal elevation in intraocular pressure (IOP) frequently associated with Primary Open Angle Glaucoma (POAG) [1], [2]. Lowering IOP delays or prevents the loss of vision in POAG patients, including in those with normal IOP that show progression and remains the only proven treatment in glaucoma [3]–[5].

Although the specific mechanisms that regulate the resistance to aqueous humor outflow in the TM/SC pathway are not completely understood [6]–[8], abundant evidence demonstrates that inhibition of the actomyosin system of the outflow pathway cells effectively increases aqueous humor drainage and lowers IOP [9]–[12]. The TM has been shown to relax or contract in response to pharmacological and biological agents due to its smooth muscle-like contractility properties [13]–[17]. Contractility of the TM is one of the potential modulators of TM conductivity and agents that induce TM contraction can reduce outflow facility [18]–[22]. Cellular contraction is believed to decrease TM permeability and aqueous humor outflow by reducing the size of the intercellular spaces, while cell relaxation will induce the opposite effect [16], [23]. In addition, alteration of the tone of TM cells induced by various factors present in the aqueous humor such as TGFβ2, lysophosphatidic acid (LPA), and endothelin 1 (ET-1) [24]–[32] have been hypothesized to contribute to the pathogenic increase in outflow resistance in glaucoma [33]–[36]. However, there is still limited information about the endogenous mechanisms regulating the contractile responses in TM cells.

MicroRNAs (miRNAs) are well recognized as important regulators of gene expression that participate in numerous normal and pathological biological processes [37], [38]. Currently, very little is known about the potential role of miRNAs on the physiology of the outflow pathway and in particular in the regulation of the tone of TM cells.

**Table 1:** Primers used for Q-PCR amplification.

Gene Symbol	FORWARD 5′-3′	REVERSE 5′-3′
ETAR	TATCCTGGCCATTCCTGAAG	TTCTCAAGCTGCCATTCCTT
ZEB2	TTCCTGGGCTACGACCATAC	TGTGCTCCATCAAGCAATTC
FHOD1	GAGGACACCACACACAATCG	TCACTGACTGCACCAGAAGG
LPAR1	ATCTTGATCCCCATCCCTTC	ACTTGCACCAAACCACACAA
ZEB1	GGAGGAGGAGGAAGAAGTGG	GCTTGACTTTCAGCCCTGTC
GAPDH	TCGACAGTCAGCCGCATCTTCTTT	ACCAAATCCGTTGACTCCGACCTT

Gene names: **ETAR**, endothelin receptor A; **ZEB2**, Zinc finger E-box-binding homeobox 2; **FHOD1**, formin homology 2 domain containing 1; **LPAR1**, Lysophosphatidic acid receptor 1; **ZEB1**, Zinc finger E-box-binding homeobox 1; **GAPDH**, Glyceraldehyde 3-phosphate dehydrogenase.

A potential regulator of the actomyosin system in TM cells is the miR-200 family. This family consists of 5 members and is believed to play an essential role in tumorigenesis and fibrosis by inhibiting cell motility and epithelial to mesenchimal transition (EMT), which have been attributed mainly to targeting of transcription factors ZEB1 and ZEB2 [39]–[42]. Recently, miR-200c has also been shown to suppress migration and invasion of cancer cells by interfering with the cytoskeletal organization through actin regulatory proteins, like FHOD1 and PPM1F, in a ZEB1/ZEB2 independent manner [43].

Our previous studies have shown that miR-200c is highly expressed in TM cells [44]. A preliminary study on mirnas induced by oxidative stress in HTM cells showed miR-200c as a highly up-regulated miRNA, and gene expression profile was analyzed after over-expressing miR-200c in HTM cells (data not published). Some genes that significantly change expressions were selected for further analysis because they were predicted targets of miR-200c and affect cell contraction. To gain insight on the role of miR-200c on contractility of the outflow pathway we investigate and identified novel target genes of miR-200c involved in the regulation of the contractile responses in TM cells, analyzed the effects of miR-200c on contraction forces exerted by TM cells, and evaluated the effects of changes in mir-200c activity on IOP in vivo.

**Table 2:** Quantitative –PCR (Q-PCR) and Affymetrix arrays values for some targets and predicted targets of miR-200c.

Gene symbol		ETAR	ZEB2	FHOD1	ZEB1	LPAR1
HTM36	fold	−1.68958	−6.9163	−2.1785	−2.45661	−1.22264
Q-PCR	p-value	0.008339	0.000162	0.000131	8.81E-05	0.027155
HTM88	fold	−1.5728	−1.84463	−1.11214	−1.54043	−1.59475
Q-PCR	p-value	0.024349	0.012577	0.175064	0.011216	0.032013
HTM23	fold	−2.92432	−4.90983	−2.28431	−3.46166	−2.31909
Arrays	p-value	0.003594	0.001477	0.005014	5.37E-05	0.000243

## Materials and Methods

### Ethics Statement

The use of animals for this study was conducted in compliance with the ARVO Statement for the Use of Animals in Ophthalmic and Vision Research. Duke University Institutional Animal Care & Use Committee (IACUC) (http://vetmed.duhs.duke.edu/IACUC.html) specifically approved this study. The Duke University School of Medicine Institutional Review Board (http://irb.duhs.duke.edu/) provided written informed consent for the original human work that produced the tissue samples.

**Figure 1:**
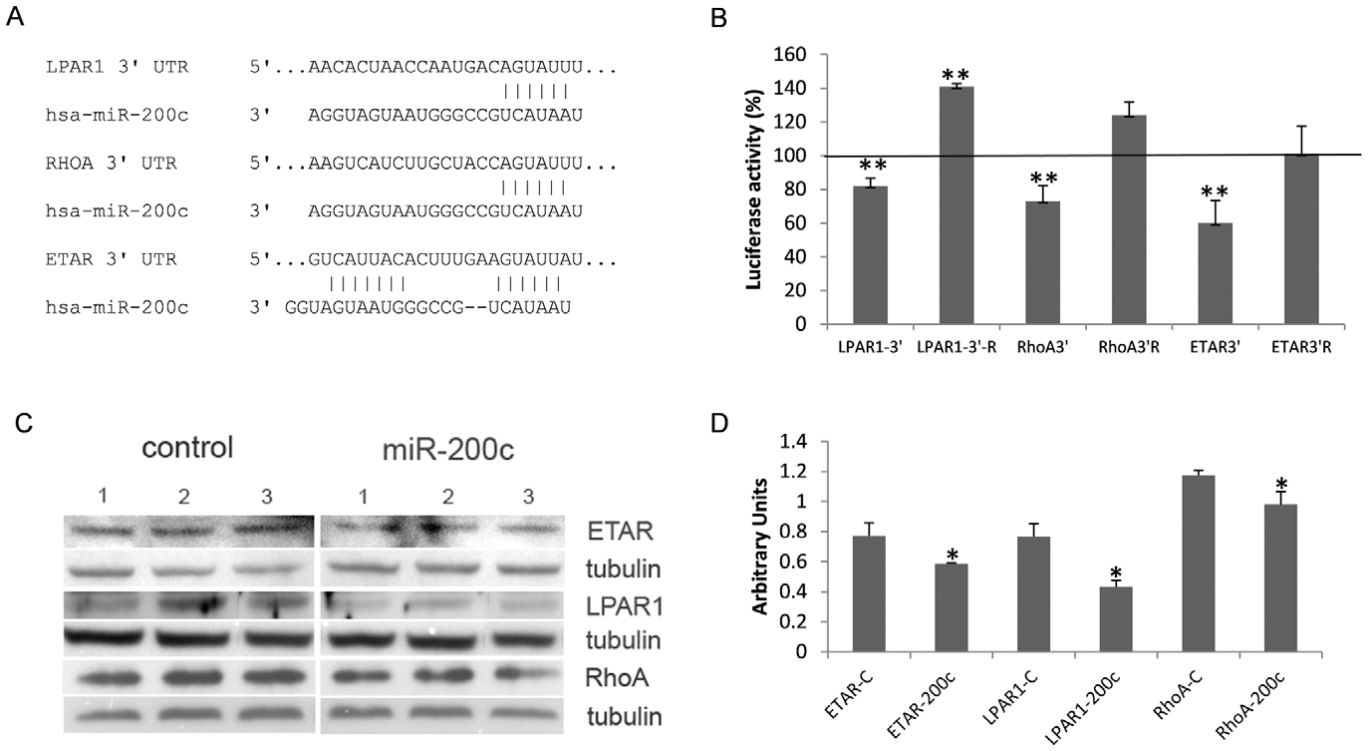
LPAR1, ETAR and RhoA are new targets of miR-200c. (A) Predicted interactions between the seed region of miR-200c and the 3′UTRs from LPAR1, ETAR and RhoA. (B) Percentage of luciferase activity in 293 cells co-transfected with psicheck vectors containing the 3′UTR or complementary sequences (R) from ETAR, LPAR1 and RhoA genes and miR-200c or miR-control. (C) Effect of miR-200c on ETAR, LPAR1 and RhoA at protein level, analyzed in HTM cell cultures by Western blot. (D) Average densitometry of proteins normalized against tubulin. Bars represent standard deviation in three different experiments. Asterisks (*) and (**) represent significant at p<0.05 and 0.01 respectively.

### Cell Culture Conditions

Human trabecular meshwork (HTM) primary cell cultures were generated from cadaver eyes, with no history of eye disease, as previously reported [45]. All procedures involving human tissue were conducted in accordance with The tenets of the Declaration of Helsinski. Human embryonic kidney 293A (HEK293A) cell line was purchased from Invitrogen (Carlsbad, CA). Cell cultures were maintained at 37°C in 5% CO_2_ in media (low glucose Dulbecco’s Modified Eagle Medium with L-glutamine, 110 mg/ml sodium pyruvate, 10% fetal bovine serum, 100 µM non-essential amino-acids, 100 units/ml penicillin, 100 µg/ml streptomycin sulfate). All the reagents were obtained from Invitrogen (Carlsbad, CA).

**Figure 2:**
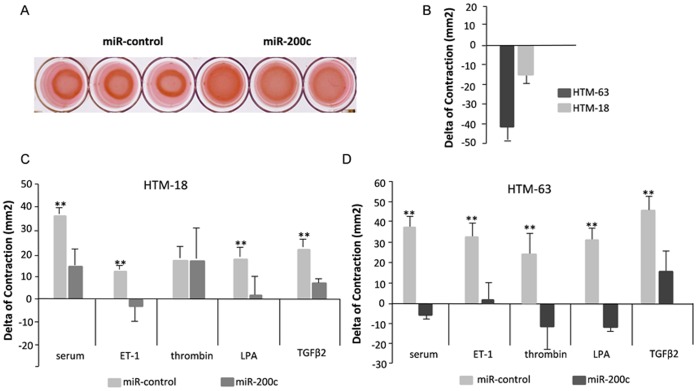
MiR-200c impairs cell contraction in collagen populated gels. (A) Representative gels of HTM cells transfected with miR-200c or miR-control in complete media. (B) Basal level of contraction for HTM63 and HTM18 was calculated as the difference in area between cell transfected with miR-control and miR-200c. The effects of miR-200c or control in the contractility response to complete media (serum) or ET-1 (200 pM), LPA (10 µM), thrombin (1 U/ml) and TGFβ2 (10 ng/ml) in serum free media, are showed in Panels C and D; in the same HTM cultures showed in Panel B. The gel area was calculated using J software. Bars represent standard deviation in three different experiments. Asterisk (**) represent significant at p<0.01 between miR-200c and control.

### RNA Isolation and Quantitative PCR (Q-PCR)

Total RNA was isolated using RNeasy kit (Qiagen Inc. Valencia, CA) according to the manufacturer’s instructions. RNA yields were measured using RiboGreen fluorescent dye (Invitrogen). First strand cDNA was synthesized from total RNA (600 ng) by reverse transcription using oligodT and Superscript II reverse transcriptase (Invitrogen) according to manufacturer’s instructions. Q-PCR reactions were performed in 20 µl mixture containing 1 µl of the cDNA preparation, 1X iQ SYBR Green Supermix (Biorad, Hercules, CA), using the following PCR parameters: 95°C for 5 minutes followed by 50 cycles of 95°C for 15 seconds, 65°C for 15 seconds and 72°C for 15 seconds. GADPH was used as internal standard of mRNA expression. The absence of nonspecific products was confirmed by both the analysis of the melt curves and by electrophoresis in 3% Super acrylAgarose gels. The primers used for Q-PCR amplification were design using Primer 3 [46] and are shown in Table 1.

**Figure 3:**
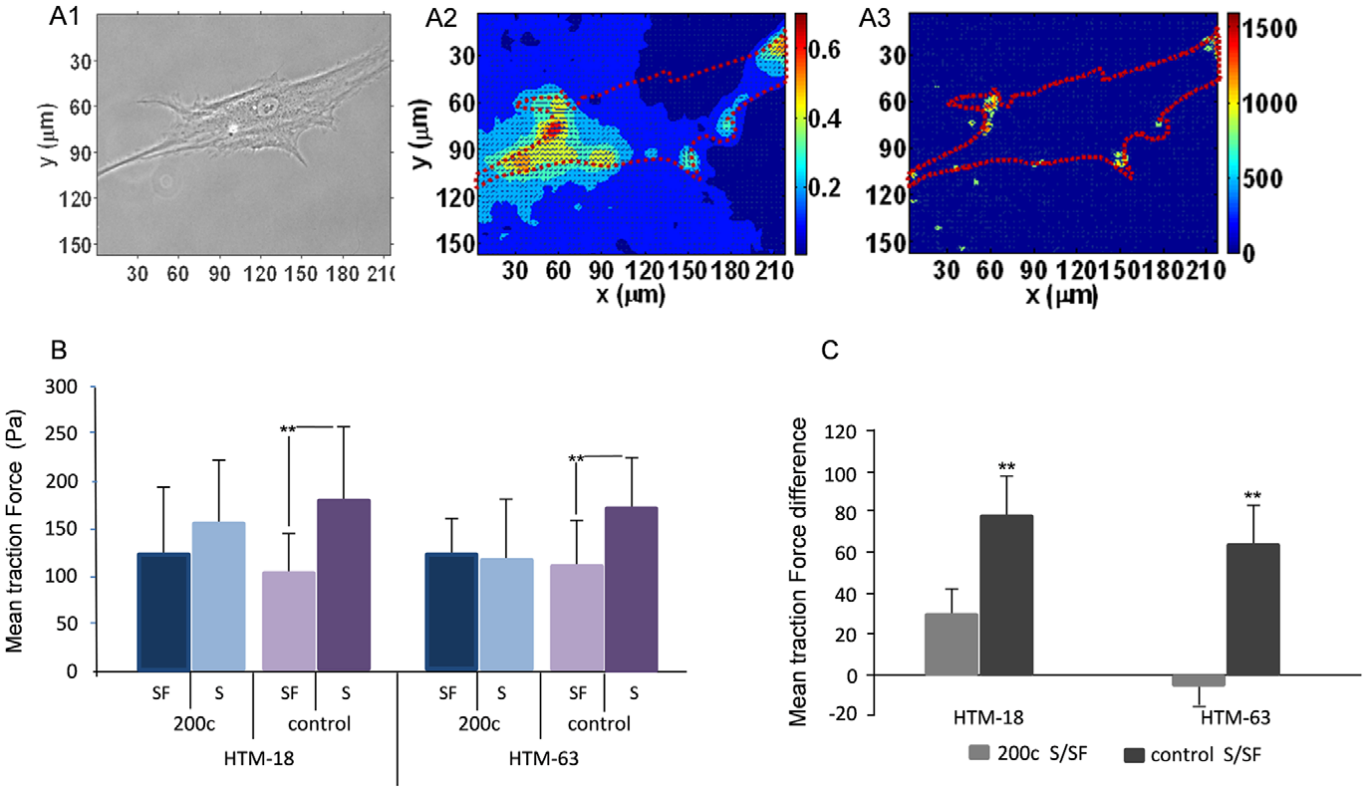
Mir-200c reduces the traction stress of HTM cells after stimulation with serum. (A1) A representative phase contrast image shows a cell on a deformable substrate, the red line depicts cell boundary from the bright field image. (A2) Displacement field of the same cell was determined by digital image correlation analysis, unit of color-bar is in µm (A3) Cell traction induce stress field was recovered from the displacement field using the method described in Huang et al.,2009; unit of color-bar is in Pascal (Pa). Color scales indicate the magnitude of displacement and traction force; X and Y represent local spatial coordinates in the projected cell area. (B) Average traction stress in two HTM primary cells (HTM18 and HTM63) transfected with miR-200c or control mimic and maintained in medium with serum (S) or serum free (SF). Panel C shows the mean difference in traction stresses between miR-200c S/SF and mimic control S/SF in HTM. HTM18 miR200c S n = 14, SF n = 15; control S n = 16, control SF n = 10; HTM63 miR-200c S n = 12, SF n = 12; control S n = 14, control SF n = 15. Bars indicate standard deviation. Asterisks (**) represent statistical significance at p<0.01.

### Transfections

HTM primary cells were transfected, at 50 to 70% confluency next day after plating, with hsa-miR-200c mimic, control mimic (scramble) or control fluorescent mimic DY547 (40 pmol) (Thermo Scientific, Chicago, IL) using lipofectamine 2000 (Invitrogen), following manufacturer’s instructions. Co-transfections in 293A cells with luciferase 3′UTR constructs (300 ng) and miR-200c mimic or scramble (20 pmol) was accomplished using Effectene (Qiagen).

**Figure 4:**
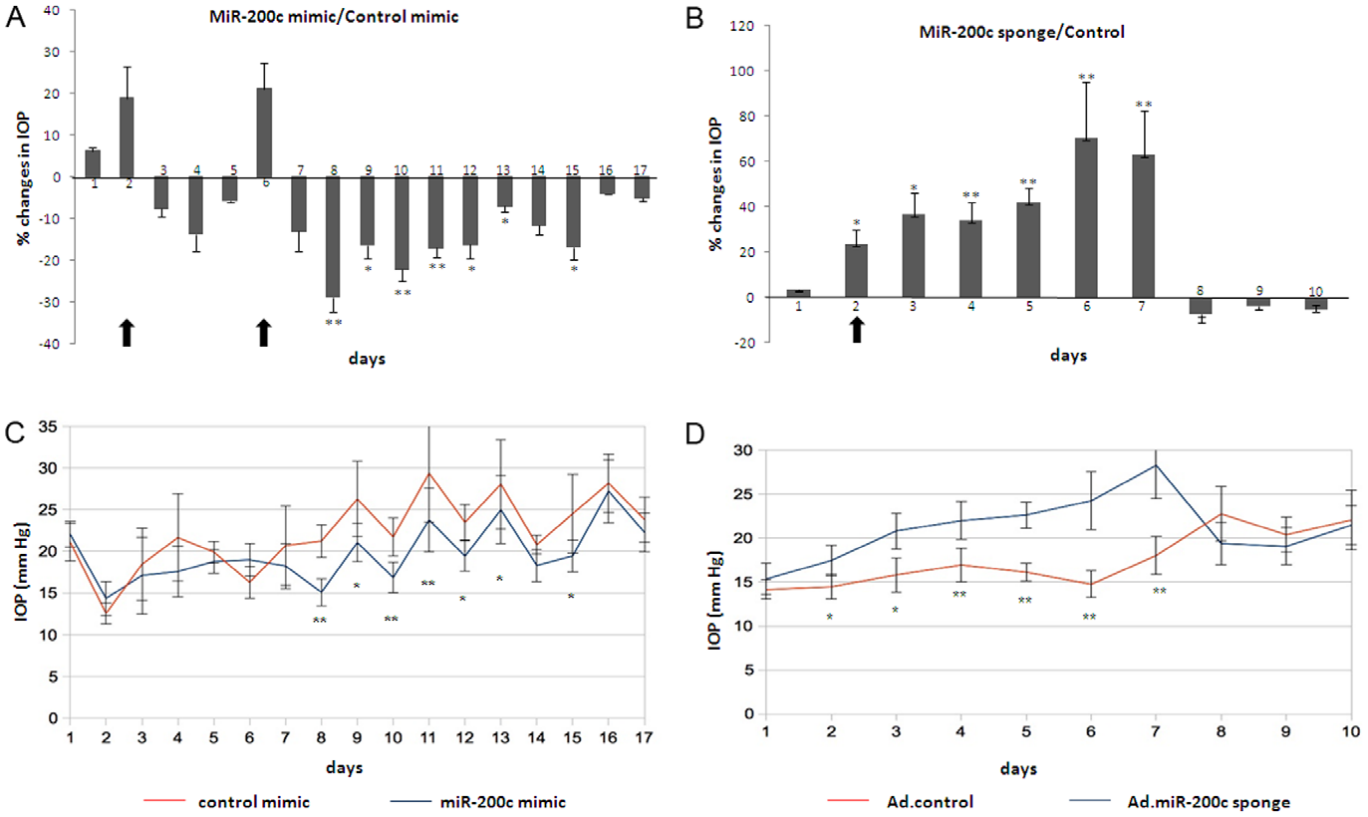
MiR-200c modulates IOP. (A) Mean IOP percent changes in rats injected with miR-200c mimic and control mimic (n = 7). (B) Mean IOP percent changes in rats injected with Ad-miR-200c sponge (1×10^9^ pfu), a decoy for miR-200c, and null adenovirus (1×10^9^ pfu) (n = 8). (C) Mean IOP values in rats injected with miR-200c mimic and control, and (D) mean IOP values in rats injected with Ad-miR-200c sponge and control. IOP was measured in the dark phase, every day. Changes in IOP were calculated as a percentage of the eye injected with experimental treatment compared to the contra lateral eye injected with control. Bars represent standard deviation. Asterisks (*) and (**) represent significant at p<0.05 and 0.01 respectively. Arrows represent injection days.

### Gene Microarray Analysis

Gene array analysis was conducted with either miR-200c mimic or mimic control on a HTM primary cell line (HTM23). Total RNA was extracted three days post-transfection using RNeasy kit (Qiagen), amplified (1 round amplification) using One cycle target labeling and control reagents (Affymetrix, Santa Clara, CA) and hybridized to Human Genome U133A2 Arrays (Affymetrix) at Duke University Microarray facility. Raw data was normalized and analyzed using GeneSpring GX10 (Silicon Genetics). Intensity-dependent normalization was performed per spot and per chip (LOWESS). ANOVA test was performed (p-values ≤0.05) for genes differentially expressed using the Benjamin and Hochberg False Discovery Rate correction test.

### Analysis of miR-200c Interaction with 3′UTRs

The entire 3′UTR from lisophosphatidic acid receptor 1 (LPAR1) and partial 3″UTR from endothelin receptor A (ETAR) and RhoA, including miR-200c complementary sites, were amplified from human sequences using the following primers LPAR1-F-gtggtttagaacggaaactg and LPAR1-R- aggtggttacttctgggttg; ETAR-F-tctagactgtctctgtggaa and ETAR-R-gccttgcaattcaagcaact; RhoA-F cgcttttgggtacatggagt and RhoA-R-gtgcagaggagggctgttag respectively, with carried XhoI and NotI restriction sites in the forward or the reverse position. PCR amplifications from 3′UTR and the complementary sequences were confirmed by sequencing and cloned into *Xho*I and *Not*I sites downstream of Renilla luciferase in the psiCheck2 vector (Promega, Madison, WI). For analysis of luciferase activity, 293A cells were seeded in 12 well plates, transfected 24 hours later with psicheck 3′UTR or the complementary sequence from LPAR1, ETAR and RhoA (300 ng each), and miRNAs for 200c mimic or control mimic (20 pmol). Luciferase was measured using the Dual Luciferase Kit (Promega, Madison, WI) following manufacturer’s instructions and read in a TD-20/20 luminometer (Turner Designs, Sunnyvale, CA).

### Protein Extraction and Western Blotting

HTM cells were transfected in triplicates, and after 72 hours washed in PBS and lysated in 1X cold RIPA. Protein concentration was determined using Micro BCA Protein Assay Kit (Pierce, Rockford, IL) and equal loading (30 µg) was run in 10–12% SDS-PAGE and transfer to PVDF membranes. Membranes were incubated overnight at 4°C, with antibodies against ETAR (Santa Cruz Biotechnology, Santa Cruz, CA), LPAR1 (Abcam, Cambridge, MA), RhoA (Cell Signaling, Beverly, MA) or tubulin (Santa Cruz Biotechnology). Blots were developed using a chemiluminescence detection system (ECL-Plus from Amersham, Buckinghamshire, UK).

### Contractility Assay and Treatments

Collagen gels were prepared in 24 well plates from rat tail collagen type 1 (1.5 mg/ml, BD Biosciences, Bedford, MA) following manufacturer’s instructions. After 24 hours transfected HTM cells were embedded in the collagen preparation before pouring, and polymerized at 37°C, 5% CO_2_ for 30 minutes. After polymerization complete media was added and gels were incubated for 48 hours before any treatment. Cells were changed to serum free media for an overnight culture and TGFβ2 (10 ng/ml), LPA (10 µM), ET-1 (200 pM) and, thrombin (1 U/ml) (all from Sigma Aldrich, St. Louis, MO) were added the next morning to serum free media. After 30 minute treatments the gels were detached from the walls and photographed 24 hours later. The gel area was calculated using Image J software [47] and transformed from arbitrary units to mm^2^. To evaluate the effects of miR200c on the levels of contraction induced by TGFβ2, LPA, ET-1, thrombin, or serum, the increase in contraction induced by each of these factors in both, cells transfected with miR-200c and cells transfected with scrambled control, was calculated as the difference in gel area between treated and non-treated cells. The basal level of contraction for each cell line in these experiments was calculated as the difference between miR-control and miR-200c (control- miR200c) in serum free media without any treatment.

### Cell Traction Force Microscopy

A solution of acrylamide (8%) and Bis-acrylamide (0.04%), was prepared with 10 mM HEPES. It was mixed with green fluorescent beads (0.2 µm) (Invitrogen) and gelled with 0.5% ammonium persulfate and 0.05% TEMED on a cover slide. The Young modulus of the gels, 17 kN/m2, was determined base on Zachary, 2006 [48]. The polyacrylamide gel was coated with collagen type 1 (BD Bioscience, Bedford, MA), sterilized with UV and incubated with media for 4 hours. Transfected cells were sparsely seeded on collagen and incubated at 37°C with 5% CO^2^, for 48 to 72 hours prior to Cell Traction Force Microscopy (CTFM). CTFM was performed under an inverted microscope (Zeiss X-cite series 120) using a method described in previous studies [49]–[51]. Briefly, images of cells and fluorescent beads were recorded both before and after cell trypzinization. The trypzinization-induced displacement of fluorescent beads in the gel was mapped, by using a gradient-based digital image correlation technique [52]. The displacement map was converted to the stress distribution, using a numerical algorithm based on the integral Boussinesq solution to generate high-resolution and applying a gradient-based digital image correlation method to track bead displacement after cell trypzinization.

### Construction of Recombinant Adenovirus

To inhibit the activity of miR-200c a sponge was used, a miRNA sponge has complementary binding sites to a miRNA of interest that can sequester miRNAs from their endogenous targets and thus serve as a decoy [53]. Replication deficient adenoviruses for miR-200c sponge and null virus were prepared using the Getaway System from Invitrogen, following manufacturing’s instructions. Briefly, miR-200c sponge was obtained through amplifying the 3′UTR from the ZEB1 gene that contain two target sites for miR-200c, using the following primers: F-ggatccgcatttcagacatggacatgctattg and R-ctcgagattaatactgccaggtttgaagacatacag. The PCR product (197 bp) was cloned in 2.1 TOPO vector (Invitrogen) and confirmed by sequencing. The 3′UTR region was release from digestion with BamHI and XhoI restriction enzymes and introduced into pENTR1A (Invitrogen). MiR-200c 3′UTR/pentr1A plasmid was recombined with pAD/CMV/V5-DEST (Invitrogen) using LR recombinase (Invitrogen) and sequencing to confirm it. pAD-CMV-miR200c-sponge was transfected to 293A cells using lipofectamine 2000. Null virus was prepared by transfecting 293 with pAD-CMV/V5-DEST. Viruses were purified and titer determined using the Adenovirus mini purification Kit (VIRAPUR, San Diego, CA) and Adeno-X Rapid Titer Kit (Clontech, Mountain View, CA) respectively.

### In Vivo Injections and IOP Measurements

Sprague-dawley male rats, 6–7 weeks old (Harlan Laboratories), were anesthetized intraperitoneally with a mixture of ketamine-xylazine (60 mg/Kg ketamine and 5 mg/Kg xylazine) plus topic anesthetic before intracameral injections in both eyes, one with control and the other with experimental treatment, using Hamilton syringes (33 gauges). Seven rats were injected with miR-200c mimic and scramble mimic (Dharmacon, 6 ug/10 ul) using The Max Suppressor In Vivo RNA-LANCER II (Bioo Scientific, Austin, TX) following manufacturer’s instructions; rats were injected twice, at days 2 and 6. Eight rats were injected with adenovirus expressing miR-200c inhibitor (miR-200c sponge, 1×10^9^ pfu, 10 µl) and with null adenovirus (empty virus, 1×10^9^ pfu, 10 µl). IOP was measured in the dark phase, every day, in animals anesthetized with inhaled isoflurane (Butler Animal Health Supply, Dublin, OH), using a portable tonometer (Tonolab, Helsinky, Finland). Relative changes in IOP were calculated as a percentage of the eye injected with experimental treatment compared to the contra lateral eye injected with control.

### Statistical Analysis

The data were presented as the mean ± SD. The significance of the data was analyzed using non-paired Student’s t-test for experiments conducted with cells, and paired Student’s t-test for the analysis of IOP in animals. A probability of less than 5% was considered statistically significant.

## Results

### MiR-200c Down-regulates Genes Involved in Cell Contraction

Gene expression profile was analyzed by gene arrays in HTM cells transfected with miR-200c or scramble control (data not showed); from a list of genes with fold change ≥2.0 or -2.0 and p-value ≤0.05 three genes ETAR, LPAR1 and FHOD1 (Table 2) were selected for further analysis because they were down regulated, predicted targets of miR-200c and affect cell contraction. ETAR is a receptor of Endothelin-1, LPAR1 is a receptor of lisophosphatidic acid and FHOD1 mediates thrombin stress fiber formation. The down-regulation of these genes was confirmed by Q-PCR in two different cell lines (HTM 36 and HTM 88), along with ZEB1 and ZEB2, two established targets of miR-200c and transcriptional repressors of E-cadherin and epithelial to mesenchimal transition [39]–[41] (Table 2). ETAR and LPAR1 are predicted as targets for miR-200c from three databases: Microcosm (http://www.ebi.ac.uk/enrightsrv/microcosm/htdocs/targets/v5/) TargetScan (http://www.targetscan.org), and PicTar-Vert (http://pictar.mdc-berlin.de/). FHOD 1 was recently described as a miR-200c target [43]. Efficiency of transfection of mirnas in HTM cells was evaluated by the reduction of miR-200c established targets (Table 2) and by fluorescent microscopy and fluorescent activated cell sorting (FACSCAN) after transfection with a fluorescent mirna (Figure S1A, S1B and S1C).

### LPAR1, ETAR and RhoA are Direct Targets of miR-200c

MiRNA databases show that miR-200c shares complementarities with sequences in the 3′UTR of ETAR, LPAR1 and FHOD1 and these genes were found to be down-regulated by miR-200c in arrays and Q-PCR analyses. RhoA is another predicted target that, although did not change in our array analyses, it was included in the investigation because of its important role in cell contraction (Figure 1A). The interaction among the 3′UTRs of ETAR, LPAR1 and RhoA with miR-200c was analyzed using the psiCheck2 luciferase assay system. MiR-200c mimic significantly reduced luciferase expression in cells co-transfected with the 3′UTR of ETAR, LPAR1 and RhoA compared to mimic control (scrambled) The decrease in luciferase activity was prevented when the 3′UTR complementary sequences were used (Figure 1B). Down-regulation of Etar, Lpar1 and RhoA proteins by miR-200c was confirmed by Western blot in HTM cells (Figure 1C and 1D).

### MiR-200c Inhibits Contraction of TM cells in Collagen Populated Gels

To evaluate the effects of miR-200c on HTM cell contraction, two primary cell cultures were transfected in triplicates, with control or miR-200c, embedded in collagen gels and the gel area was measured at 24 hours after gel detachment. Cell contraction was assessed in complete media, media without serum and media without serum and ET-1, LPA, thrombin or TGFβ2. These factors were chosen because they are known to induce contraction of trabecular meshwork, and miR-200c targeted main receptors for ET-1 and LPA (ETAR and LPAR1), and a gene inducing stress fiber formation by thrombin activation (FHOD1). Figure 2A showed the difference in contraction between cell transfected with control and miR-200c for each cell line. The difference in contraction induced by serum, ET-1, LPA, thrombin or TGFβ2 is showed in Figures 2A, 2C and 2D. Cells transfected with miR-200c exhibited a significant decrease in contractility in response to serum, LPA, ET-1 and TGFβ2 compared to controls. On average the cells in complete media showed the biggest difference in contraction between miR-200c and scramble (28.8 mm^2^). In some instances miR-200c abolished almost completely the contractile response to these treatments (Figure 2C and 2D).

### MiR-200c Reduces Traction Force in HTM cells after Stimulation with Serum

Cell traction force plays a key role in many physiological and pathological processes through actomyosin interactions. The approach to measure cell traction forces is based on the use of flexible polyacrylamide coated with extracellular matrix protein for cell adhesion and embedded with fluorescent beads for tracking the deformation as a result of exerted forces by individual cells [54]. In order to analyze the effects of miR-200c on the traction force of HTM cells we measured stress distribution using CTFM technique in cells transfected with miR-200c or control with and without serum (Figure 3A). In complete media, cells transfected with miR-200c exhibited less mean traction force than cells with miRNA control; this difference was reduced when cells were kept in media without serum. The average difference between mean traction force in cells with and without serum in the control group was statistically significant; but in cells transfected with miR-200c there was no significant difference (Figure 3B and 3C).

### MiR-200c Modulates IOP in vivo

To investigate if miR-200c could be relevant for regulation of intraocular pressure *in vivo*, rats were injected intracamerally with miR-200c mimic, control mimic, Ad-miR-200c-sponge and Ad-empty virus. The efficacy of miR-200c sponge was confirmed by Q-PCR in HTM cells (Figure S2B). Following injections there was no evidence of inflammation or redness in the eyes. The eyes injected with mir-200c mimic showed a reduction in IOP compared to the contra-lateral eye injected with control mimic, the maximum difference between the two eyes was observed at day 8 with average IOP of 21.2 mmHg in the control and 15.1 mmHg in the experimental. The reduction in IOP was significant during seven days but just after a second injection of miRNA on Day 5, and this reduction effect lasted for 9 days. Eyes injected with Ad-miR200c-sponge showed a significant increase in IOP compared to the contra-lateral eye injected with empty virus. The maximum difference between both eyes was observed at day 6 with average IOP of 14.7 mmHg in the control and 24.2 mmHg in miR-200c sponge, this effect was statistically significant during the 6 days that the effect lasted (Figure 4).

## Discussion

Our results showed that cells transfected with miR-200c down-regulated ZEB1, ZEB2, FHOD1, LPAR1/EDG2, ETAR; the same treatment also down regulated RhoA at protein level. FHOD1 was recently described as a target of miR-200c; and here we identified ETAR, LPAR1/EDG2, and RhoA as novel targets of miR200c as well. All these proteins play roles potentially relevant to the regulation of the tone of the TM cells. ZEB1 and ZEB2 regulate the expression of SMA that is known to be induced by TGFβs in HTM cells [55], [56]. FHOD1 and RhoA are well known activators of the actomyosin system [57], [58]. ETAR is one of two major ET-1 receptors and mediates the increase in intracellular calcium and vasoconstriction induced by ET-1. While the second type of ET-1 receptor, ETBR, frequently counter-regulates ETAR activity through production of nitric oxide and ET-1 clearance, ETAR mediates the ET-1 induced contraction of cells, including TM cells [25], [28], [35], [59]–[63]. The physiological agonist of LPAR1, LPA, has been demonstrated to increase cell contraction of cells in the outflow pathway [33], [34].

Consistent with the observed postranscriptional inhibition of multiple genes involved in the regulation of the contractile responses, HTM cells transfected with miR-200c showed a decreased response in cell traction forces. Cell traction forces direct many cellular functions, as cell migration, ECM organization, and generation of mechanical signals. Mir-200c reduced contractile forces in population of cells and in some cases almost abolished the contractile responses induced by ET-1, LPA, and TGFβ2. In HTM cells LPA, TGβ2 and RhoA have been shown to induce alpha-SMA [55] and ET-1 increased alpha-SMA in pulmonary fibroblast [64]. Alpha-SMA has been shown to up-regulate cell traction force in myofibroblasts in a direct fashion [65], [66]. Furthermore, transfection of HTM cells with miR-200c significantly decreased traction forces in single cells in presence of serum. Serum contains a complex mixture with growth factors, cytokines, and enzymes, and it is known to induce cell contraction in several cell types [67]–[69]. Serum includes multiple factors present in the aqueous humor that can induce trabecular contraction such as ET-1, LPA and TGFβ2 [24]–[32].

ET-1, LPA and TGFβ2 have been implicated in pathogenic increase in tissue stiffness and aqueous outflow resistance observed in glaucomatous TM [70]. These factors present in the aqueous humor have been proven to exert significant effects on outflow facility. ET-1 has been related to alterations in IOP, ocular blood flow, optic nerve integrity and retinal ganglion cell survival [35]. Increased presence of ET-1 in the AH is believed to contribute to the pathogenesis of the outflow pathway in eyes with POAG [28], [60], [61], [63] and inhibition of ET-1 signaling is considered to be one of the new potential approaches to the treatment of glaucoma [62], [71]. The potential role of ET-1 in glaucoma might result from two different mechanisms: (1) increased vasoconstriction causing a decrease in ocular supply to the retina and the optic nerve head, and (2) IOP elevation as a result of an increase in TM cell contraction [63]. In addition, ET-1 is believed to exert pathogenic effects by inducing the production of reactive oxygen species through an ETAR dependent mechanism [59]. LPA and thrombin has been shown to reduce outflow facility in a porcine ex-vivo model and to increase stress fiber formation and MLC phosphorylation in Schlemm’s canal cells [33].

The potential role of LPAR1 in outflow pathway is particularly interesting in light of the recent observation that autotaxin, an enzyme that generates LPA, is elevated in AH from POAG donors, and its inhibition decreases significantly the IOP in rabbits [72]. Finally, TGFβ2 have been found to be elevated in the aqueous humor of glaucomatous eyes compared to age matched controls [73]; TGFβ2 can stimulate secretion of extracellular matrix factors [74], [75], induce cross linking actin networks (CLAN) formation in TM cells [76] and induce endothelin-1 synthesis in HTM [36]. In addition, expression of a constitutively active form of TGFβ2 in the TM of mice and rats is known to elevate IOP and reduce outflow facility [77].

The observed effects of miR-200c on the responses induced by ET-1, LPA and TGFβ2 suggest that miR-200c might exert important effects on IOP. MiR-200c injected into the anterior chamber of rat eyes caused a significant decrease on IOP. This effect was accumulative since it was higher after two injections of miR-200c mimic. It also appeared to last longer than the short life of miR-mimics, suggesting that some of the changes induced by the increased miR-200c expression might persist for some time after miR-200c has returned to basal levels. The eyes injected with either miR-200c mimic or control showed a larger level of IOP fluctuations than those treated with adenoviral vectors, suggesting that the method of delivery could potentially influence IOP stability. Testing and optimizing more effective methods for delivery of miRNAs to the cells of the outflow pathway will be an important objective for future studies aimed at analyzing the functional effects of miRNAs in the outflow pathway and evaluate their therapeutic potential as IOP lowering agents. The effects of miR-200c on IOP were further supported by the increase in IOP observed after inhibition of miR-200c using an adenoviral vector expressing a molecular sponge. Surprisingly, the IOP returned to normal values only 6 days after viral delivery. This decrease in IOP appeared to occur too early to be attributable to silencing of the CMV promoter activity which has been reported to occur at least a few weeks after viral delivery in multiple organs [78].

Therefore, other unidentified mechanisms may be responsible for the observed recovery in IOP. Similarly, it is worth mentioning that microRNAs are known to have multiple targets, and it is likely that additional miR-200c targets might be involved in the observed effects of this miRNA on outflow facility. Further studies will be needed to fully understand the mechanism behind the effects of miR-200c *in vivo*.

In conclusion, our results demonstrate for the first time the ability of a miRNA to regulate trabecular contraction and modulate IOP *in vivo*, making miR-200c worthwhile candidate for exploring ways to alter trabecular contractility with therapeutic purposes in glaucoma.

## Supporting Information

Figure S1 **HTM transfection Efficiency.** HTM cells were transfected using lipofectamine with a fluorescent miRNA (DY547) and analyzed 48 hours after transfection. Panel A. Light microscopy image of HTM cells. Panel B. Fluorescent image of the same field, red is fluorescent miRNA, and blue are nuclei counterstained with DAPI (1 mg/ml) (original magnification ×100). Panel C. Fluorescent activated cell sorting (FACS) analysis of HTM cells transfected with fluorescent miRNA.
(TIF)

Figure S2 **Alignment of human and rat miR-200c sequences and evidence of functionality of the miR-200c sponge.** Panel A shows miR-200c pre-mirna sequences for Rattus novergicus (Rno-miR-200c; NCBI Reference seq: NR_031915.1) and Homo sapiens (hsa-miR-200c; NCBI Reference seq: NR_029779.1) and the miR-200c sequence used as mimic; the mature miRNA is highlighted in red. (B) MiR-200c sponge activity was analyzed by Q-PCR in HTM cells transduced with miR-200c sponge or control virus (10^7^ pfu) after three days of infection. Bars represent standard deviation. Asterisks (*) and (**) represent significant at p<0.05 and 0.01 respectively.
(TIF)
