# Supplementary figures and images for: Regulation of Trabecular Meshwork Cell Contraction and Intraocular Pressure by miR-200c
Source: PLoS One. 2012 Dec 14;7(12):e51688. doi: 10.1371/journal.pone.0051688 (PMC3522713; doi:10.1371/journal.pone.0051688)

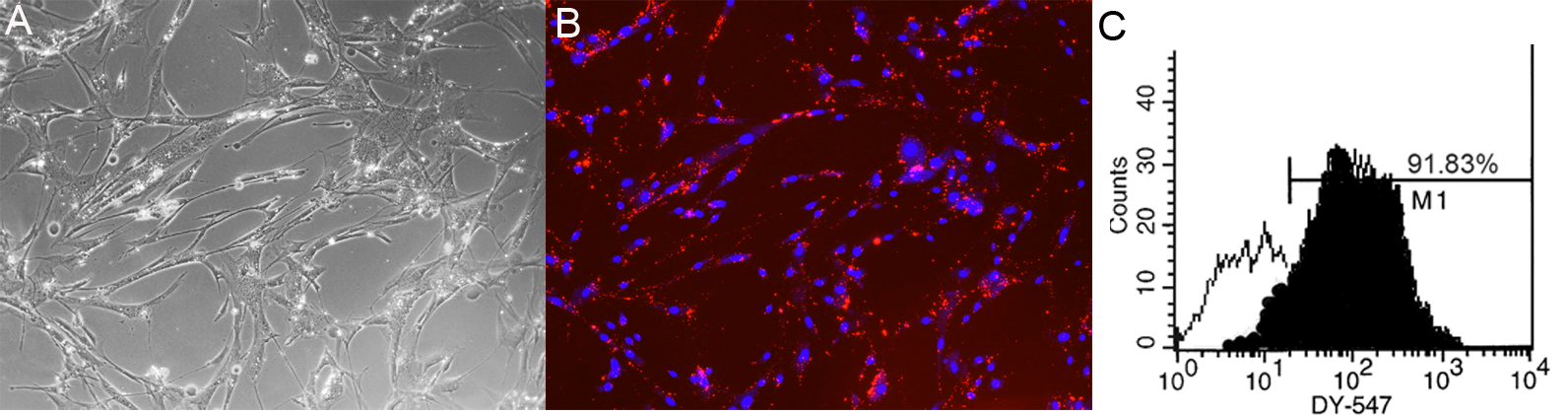

Supplement: Figure S1 — HTM transfection Efficiency. HTM cells were transfected using lipofectamine with a fluorescent miRNA (DY547) and analyzed 48 hours after transfection. Panel A. Light microscopy image of HTM cells. Panel B. Fluorescent image of the same field, red is fluorescent miRNA, and blue are nuclei counterstained with DAPI (1 mg/ml) (original magnification ×100). Panel C. Fluorescent activated cell sorting (FACS) analysis of HTM cells transfected with fluorescent miRNA. (TIF) [file pone.0051688.s001.tif]

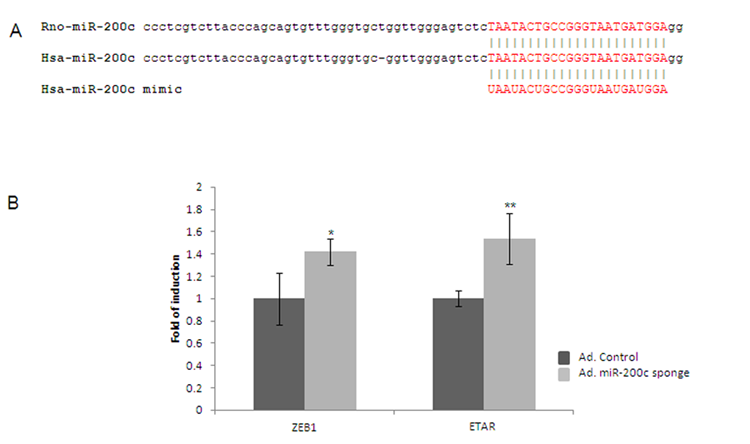

Supplement: Figure S2 — Alignment of human and rat miR-200c sequences and evidence of functionality of the miR-200c sponge. Panel A shows miR-200c pre-mirna sequences for Rattus novergicus (Rno-miR-200c; NCBI Reference seq: NR_031915.1) and Homo sapiens (hsa-miR-200c; NCBI Reference seq: NR_029779.1) and the miR-200c sequence used as mimic; the mature miRNA is highlighted in red. (B) MiR-200c sponge activity was analyzed by Q-PCR in HTM cells transduced with miR-200c sponge or control virus (107 pfu) after three days of infection. Bars represent standard deviation. Asterisks (*) and (**) represent significant at p<0.05 and 0.01 respectively. (TIF) [file pone.0051688.s002.tif]
